# Internally Harmonic Matched Compact GaN Power Amplifier with 78.5% PAE for 2.45 GHz Wireless Power Transfer Systems

**DOI:** 10.3390/mi15111354

**Published:** 2024-11-06

**Authors:** Caoyu Li, Ziliang Zhang, Yi Pei, Changchang Chen, Gang Feng, Yuehang Xu

**Affiliations:** 1 Yangtze Delta Region Institute (Huzhou), University of Electronic Science and Technology of China, Huzhou 313001, China; licaoyu@csj.uestc.edu.cn (C.L.); 202221020901@std.uestc.edu.cn (Z.Z.); fenggang@uestc.edu.cn (G.F.); 2 School of Electronic Science and Engineering, University of Electronic Science and Technology of China, No. 2006, Xiyuan Ave., West Hi-Tech Zone, Chengdu 611731, China; 3 Dynax Semiconductor Inc., Suzhou 215300, China; yi.pei@dynax-semi.com (Y.P.); changchang.chen@dynax-semi.com (C.C.); 4 National Key Laboratory of Wireless Communications, University of Electronic Science and Technology of China, No. 2006, Xiyuan Ave., West Hi-Tech Zone, Chengdu 611731, China

**Keywords:** GaN HEMT, power amplifiers, high efficiency, 2.45 GHz

## Abstract

In this paper, a high-efficiency compact power amplifier is designed and fabricated with a 0.25 μm GaN high electron mobility transistor (HEMT) to meet the demands of a high integration level and high efficiency for microwave wireless power transfer (WPT) systems. The proposed power amplifier (PA) is implemented using an internally matched method to achieve a compact circuit size. The output second and third harmonic impedances can be optimized through output matching circuits, eliminating the need for additional harmonic matching networks. This approach simplifies the design of matching circuits and reduces the circuit size. Furthermore, the input third harmonic has been controled for improving the efficiency of DC-to-RF conversion. The total size of the proposed PA is 13.4 × 13.5 mm^2^. The test results obtained from the continuous wave (CW) testing indicate that the output power of the power amplifier at 2.45 GHz reaches 43.75 dBm. Additionally, the large-signal gain is measured at 15.75 dB, and the power-added efficiency (PAE) achieves a value of 78.5%.

## 1. Introduction

Wireless power transfer (WPT) systems represent a promising trend in the future development of electronic power delivery methods [1]. The dimensions of the circuit are also critical, as the high integration level required for beam-forming structures necessitates a compact design for power amplifiers. Consequently, compact, high-efficiency, and high-power amplifiers play a vital role in WPT systems. The most crucial metric for evaluating a WPT system is its transfer efficiency. Gallium nitride (GaN) high electron mobility transistors (HEMTs) are particularly well-suited for applications demanding high power and efficiency due to their exceptional electrical characteristics.

The GaN power amplifier can be realized through three primary methods: a Monolithic Microwave Integrated Circuit (MMIC), an external matching circuit (EMC), and an internally matched circuit. In comparison to an MMIC and EMC, the internally matched power amplifier demonstrates significant advantages in terms of device performance, size, and production cost [2]. To satisfy the design requirements for miniaturization, high efficiency, and high output power, the internally matched power amplifier has increasingly become a focal point of research and has found widespread applications in satellite navigation and radar detection fields [3,4,5].

The theoretical drain efficiency (DE) of class E and class F power amplifiers is 100% [6,7,8]. Both design methodologies can satisfy the requirement for high efficiency at a single frequency. However, a significant limitation of class E power amplifiers is that the output capacitance of the transistor has a pronounced effect on the maximum operating frequency, particularly in the microwave range [9]. The theoretical DE of class F power amplifiers can also reach 100%, but this is contingent upon achieving impedance matching across all harmonics. Typically, impedance matching for the second and third harmonics necessitates additional circuit design; however, it should be noted that any insertion loss introduced by higher harmonic matching circuits may adversely affect power-added efficiency (PAE) more than the efficiency gains achieved through such matching [10].

In the microwave frequency range, a power amplifier (PA) designed with class F architecture is recognized as one of the most effective methodologies for enhancing the power-added efficiency (PAE) of narrow-band power amplifiers. Recent years have witnessed extensive research focused on harmonic termination techniques aimed at developing high-efficiency power amplifiers [11]. To meet the harmonic impedance requirements of a class F power amplifier, it is essential to manage not only the output harmonic impedance but also the input harmonic impedance. Various techniques have been proposed for harmonic tuning, including open-ended stubs integrated into external matching circuits [2,12,13], on-chip LC tuning circuits [14,15,16], and off-chip LC tuning circuits [17,18,19].

In 2009, F. M. Ghannouchi proposed a 2.45 GHz inverse class F power amplifier (PA) with a power-added efficiency (PAE) of 71.5% [20]. A. A. Ismail reported a 2.45 GHz class F PA characterized by high linearity; however, its PAE is only 62% [21]. Both of these studies employed externally matched circuits, which tend to occupy larger physical sizes compared to internally matched PAs. In 2018, Takumi Sugitani et al., from Mitsubishi Electric Corporation [14], presented a high-power and high-efficiency power amplifier designed for microwave heating applications, achieving an output power exceeding 57 dBm and demonstrating a drain efficiency (DE) greater than 70% at the frequency of 2.45 GHz. This internally matched power amplifier incorporates an on-chip LC tuning circuit situated near the gate terminals of the HEMT to regulate the input second harmonic impedance while utilizing an open-ended stub to manage the output second harmonic impedance. The on-chip LC tuning circuit offers minimal area occupation along with superior consistency in both amplitude and phase; however, it lacks tunability.

To address the growing demand for compact and high-efficiency power amplifiers in microwave wireless power transfer systems, we have designed an internally-matched power amplifier that operates with high efficiency at a frequency of 2.45 GHz, utilizing 0.25 μm GaN HEMT technology. The implementation of LC components is achieved through bonding wire and thin-film circuit techniques to ensure a compact design. Additionally, harmonic tuning methods are employed to enhance overall efficiency.

## 2. Overall Design

**Figure 1:**
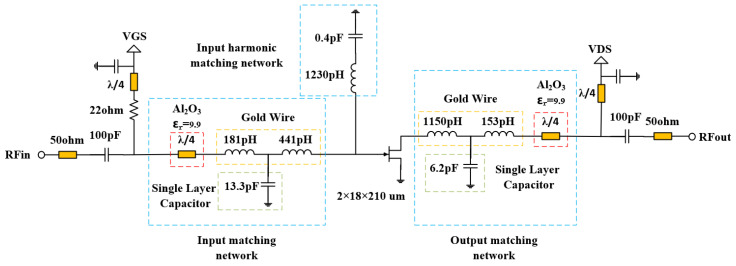
Schematic diagram of the internally matched power amplifier circuit topology.

The GaN HEMT under consideration is fabricated utilizing an advanced GaN chip production line provided by Dynax Semiconductor Inc. This device achieves a typical power density of 6.0 W/mm, a drain efficiency (DE) of 73% at 6 GHz, and a typical gain of 19.0 dB. To meet the power requirement of 20 W while ensuring high power-added efficiency (PAE) with a compact internally matched power amplifier (PA), a single-stage topology has been employed. The overall gate size of the GaN HEMT die measures 655 × 2725 μm. The schematic diagram illustrating the internally matched power amplifier circuit topology is presented in Figure 1.

In order to meet the output power requirements and maximize the power-added efficiency (PAE) of the power amplifier, the transistor operates in deep class AB mode. The gate voltage (Vgs) is set at −2.7 V, while the drain voltage is maintained at 28 V, resulting in a quiescent current of 50 mA for the transistor. Input and output matching are achieved through an LC tuning circuit that utilizes a parallel plate capacitor along with bonding wire as inductance elements. Both input harmonic matching and output harmonic matching are considered and analyzed as follows.

## 3. High-Efficiency Matching Circuit Design

Firstly, a simulation of load pull and source pull for the fundamental frequency is conducted at 2.45 GHz for the GaN HEMT die by employing an accurate model [22]. This large-signal model provides precise impedance values while taking into account the influence of the knee voltage characteristic of GaN HEMTs. The fundamental output impedance of the GaN HEMT is measured to be 5.7 + j*10.2 Ω, whereas its input impedance is determined to be 1.1 + j*3 Ω. Regarding amplifier bandwidth issues, according to the Bode-Fano criterion, there exists a constraint between the bandwidth and reflection coefficient as follows [23]:

(1) $$BW=\frac{\pi \omega_{0}}{R\ln\left(\frac{1}{\tau_{\min}}\right)}$$

(2) $$n={\left(\frac{R_{L}}{R_{S}}\right)}^{\frac{1}{K}}$$

In which, $R_{L}$ denotes the load impedance, K stands for the number of nodes in the T-type network, and n represents the impedance conversion ratio. From (1) and (2), increasing the LC network’s order will expand the bandwidth, but it will also result in greater physical space requirements and increased insertion loss. Since the power amplifier required to be designed is a high-efficiency power amplifier operating at 2.45 GHz, it is necessary to minimize the influence of the working bandwidth on the output power and PAE. So, the output matching circuit only needs to use an L-C-L network to implement the matching of the virtual part, while increasing the impedance of the real part to 10 Ω, and then transforming the real part to 50 Ω through the quarter-wavelength transformation line. The input matching circuit also uses an L-C-L network and quarter-wavelength transformation line to achieve the best match with the source impedance between 1.1 + j*3 Ω and 50 Ω.

**Figure 2:**
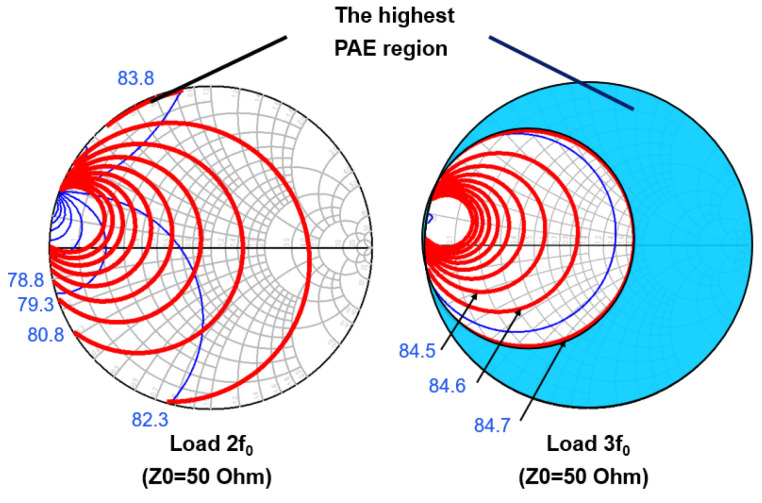
Simulated PAE dependence with the output harmonic impedance.

Second, the GaN HEMT undergoes 2nd and 3rd load/source pull simulations using precise modeling techniques. Figure 2 shows the 2nd and 3rd load pull simulation results. In Figure 2, the impedance point of the optimal PAE is offset compared with the ideal class F power amplifier due to the influence of the parasitic parameters of the transistor.

The class F power amplifier requires that the input 2nd and 3rd harmonic impedance is in the short state. The 2nd source pull simulation results show that the 2nd harmonic optimal source impedance is 1.4 + j*0.595 Ω. The 3rd source pull simulation results show that the 3rd harmonic optimal source impedance is 1.41 + j*1.79 Ω. The impedance point of the optimal PAE is slightly offset compared with the ideal class F power amplifier due to the influence of the parasitic parameters of the transistor. In order to achieve optimal harmonic source impedance, we need to increase the off-chip harmonic tuning circuit to achieve this requirement. Due to the accuracy of L and C values, the harmonic tuning circuit may cause a frequency mismatch of fundamental frequency. To avoid power and gain loss at operation frequency and to simplify the input matching network, only the 3rd harmonic tuning circuit is applied in the input matching network. The input harmonic matching network is shown in Figure 1. The 3rd harmonic tuning circuits are LC series resonant circuits, which are realized in the same way as the fundamental frequency matching circuit. The resonant frequency of a series L-C resonant circuit can be calculated by (3).

(3) $$f=\frac{1}{2\pi \sqrt{LC}}$$

**Figure 3:**
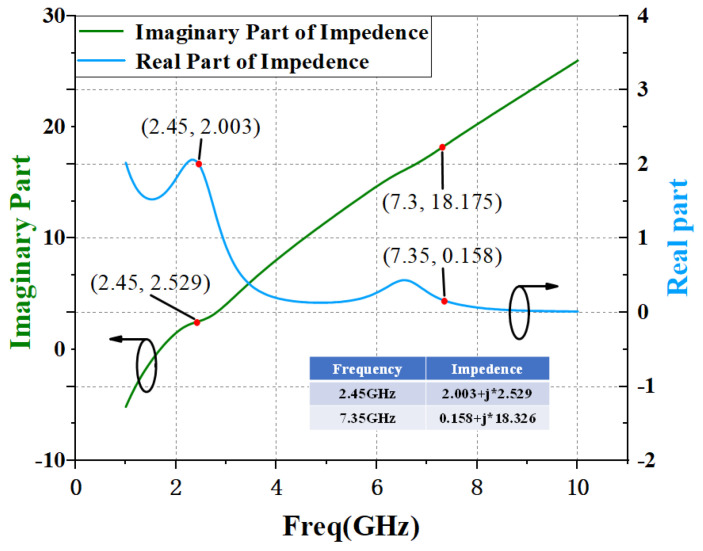
Simulation results of impedance curve of input matching circuit without harmonic tuning circuit.

**Figure 4:**
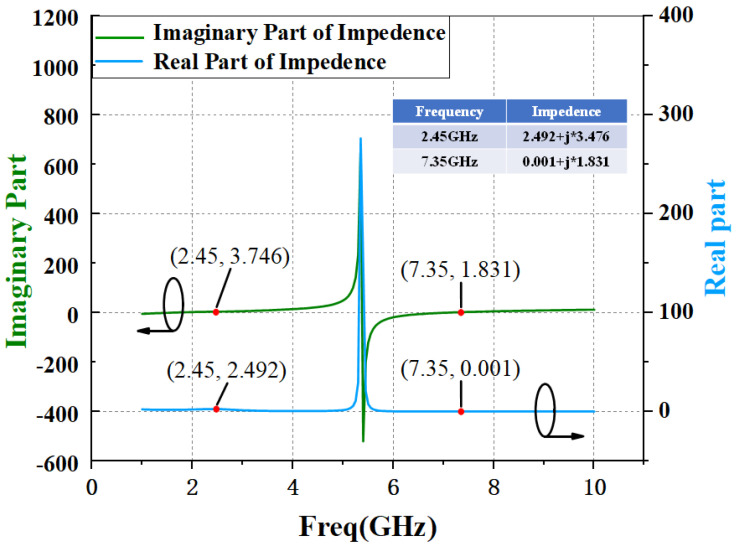
Simulation results of impedance curve of input matching circuit with harmonic tuning circuit.

By setting the values of L and C reasonably, the resonant frequency of the LC resonant circuit is the third harmonic frequency, respectively, so as to realize the short circuit state of the input third harmonic. The simulation curve of input matching network impedance with and without harmonic tuning is given by Figure 3 and Figure 4.

**Figure 5:**
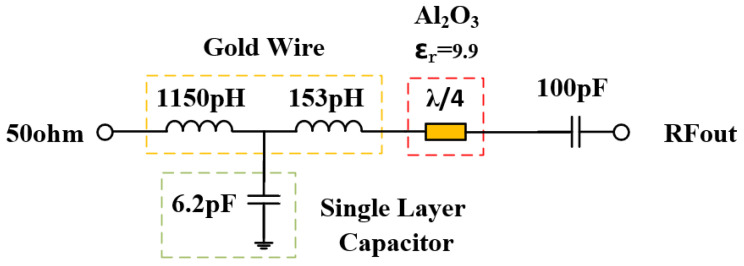
Schematic diagram of output matching circuit.

**Figure 6:**
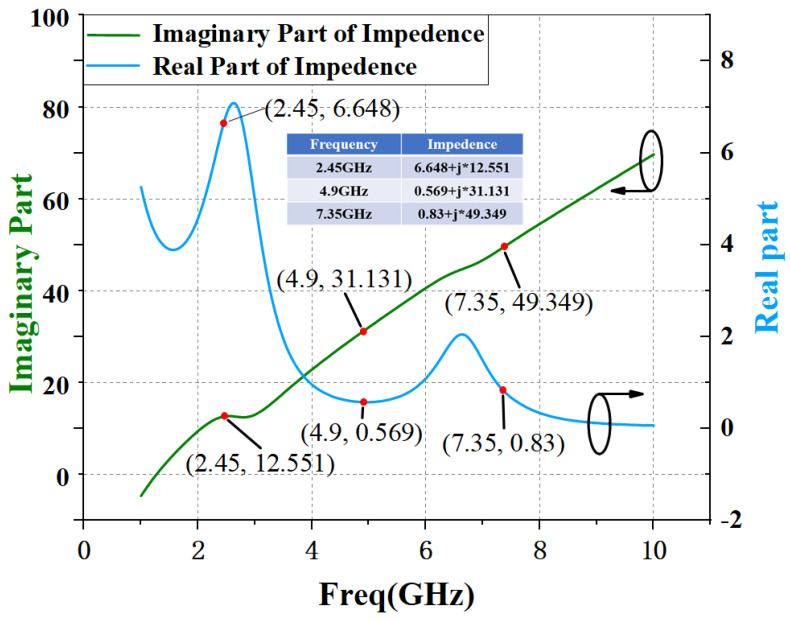
Impedance curve simulated result of output matching circuit.

Figure 5 gives the schematic diagram of the output match network. The simulation results of the impedance curve of the output matching circuit are given by Figure 6. It can be seen that the 2nd harmonic impedance achieved by the output matching circuit without harmonic tuning is 0.511 + j*31.466 Ω. If harmonic tuning is added, the 2nd harmonic impedance achieved by the output matching circuit is 0.002 + j*2.003 Ω. Combined with the 2nd load pull simulation results in Figure 2, it can be seen that the output matching circuit without the output 2nd harmonic tuning will transform the 2nd harmonic impedance to the region with the highest efficiency, while the output matching circuit with the output 2nd harmonic tuning will transform the 2nd harmonic impedance into the “trap” with low efficiency. The case of the 3rd harmonic is the same as the case of the 2nd harmonic. Therefore, for output matching, we adopt the method of combining fundamental frequency matching with the 2nd and 3rd harmonic matching, rather than the harmonic tuning method.

**Figure 7:**
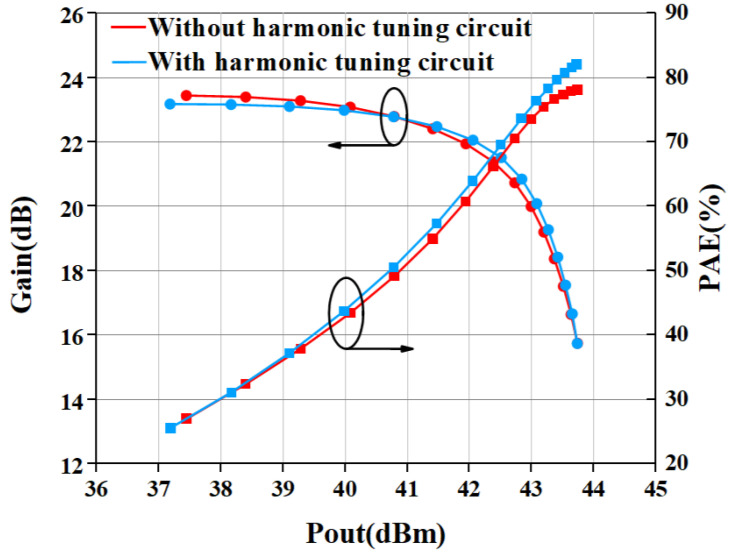
Simulated result of large-signal performance with and without harmonic tuning circuit.

Figure 7 shows the simulated result of large-signal performance with and without a harmonic tuning circuit. The output power and gain at saturation in the two cases have almost no difference. The biggest difference in PAE between the with and without harmonic tuning cases is 4% when the PA saturates.

The impedance transformation for fundamental frequency matching is achieved using an alumina substrate which has a permittivity of 9.9 and the thickness of the substrate is 0.254 mm, with a characteristic impedance of 24.1 Ω. These matching circuits are designed to realize the optimal output harmonic impedance.

The components in the matching circuit are realized by different substrate materials, and then all the components are glued to the inside of the tube shell by conducting resin, and finally connected by gold wire. The inductance L is achieved using a bonding wire with a diameter of 25 µm. Capacitor C is implemented using thin-film technology, and the substrate material is made of alumina ceramics with a relative dielectric constant of 9.9. The capacitance value of the parallel plate capacitor can be calculated by (4).

(4) $$C=\varepsilon_{0}\varepsilon_{rd}\frac{A}{d}=\varepsilon_{0}\varepsilon_{rd}\frac{W\cdot l}{d}$$

Here, *W* denotes the width of the capacitor, and *l* denotes its length. The $\varepsilon_{0}$ represents the permittivity of the medium of the capacitor, and $\varepsilon_{rd}$ represents the permittivity of the vacuum.

## 4. Results and Discussion

**Figure 8:**
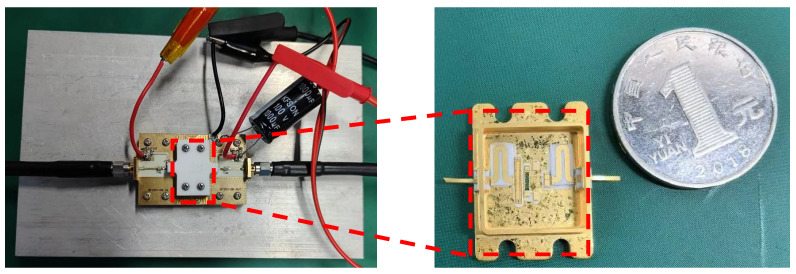
Picture of the GaN power amplifier using the harmonic tuning circuit and measurement fixture.

**Figure 9:**
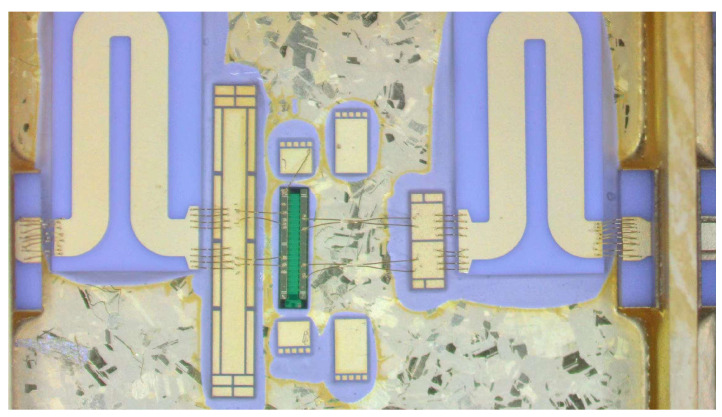
Picture of the detail of the proposed internally matched power amplifier.

We evaluated the RF performance of an internally-matched power amplifier with the measurement fixture. Figure 8 is the top-view picture of the GaN power amplifier using a harmonic tuning circuit and measurement fixture. The detail of the proposed PA is given with an enlarged photo shown in Figure 9. The metal cavity underneath the measurement fixture is used to improve the heat dissipation of the internally-matched power amplifier. The width of a single gate finger is 210 μm. The package size is 13.4 × 13.5 mm^2^. The internally-matched power amplifier is tested under the condition of a CW signal. The drain bias voltage is 28 V, and the quiescent drain current is 50 mA.

We assessed the RF performance of an internally matched power amplifier using a measurement fixture. Figure 8 presents a top-view image of the GaN power amplifier, which incorporates a harmonic tuning circuit and measurement fixture. Detailed information regarding the proposed power amplifier is provided in an enlarged photograph shown in Figure 9. The metal cavity located beneath the measurement fixture serves to enhance heat dissipation for the internally matched power amplifier. Each gate finger has a width of 210 μm, and the package dimensions are 13.4 × 13.5 mm^2^. The internally matched power amplifier was evaluated under continuous wave (CW) signal conditions, with a drain bias voltage set at 28 V and a quiescent drain current of 50 mA.

### 4.1. Small-Signal Measurement

**Figure 10:**
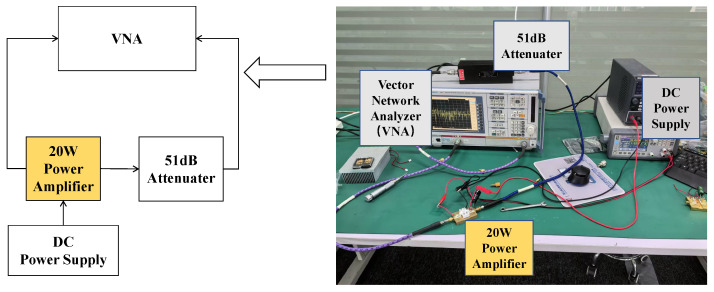
Small-signal parameter measurement setup.

**Figure 11:**
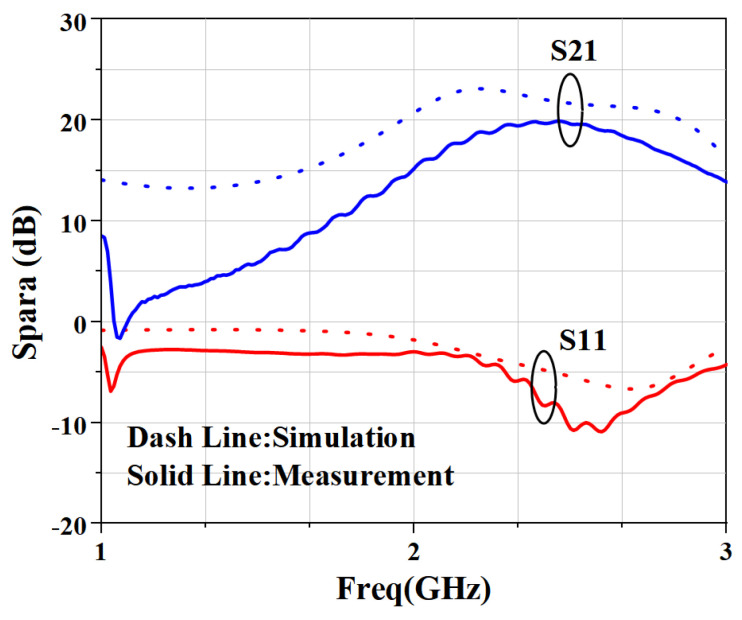
Measured and simulated S-parameter results.

Figure 10 is the picture of the small-signal parameter measurement setup. Vector Network Analyzer ZVB 8 from Rohde & Schwarz is applied to measure the small-signal parameters. A 51 dB attenuator is applied to prevent the overpowering of the Vector Network Analyzer. Figure 11 presents a comparison between simulated and measured small signal results from 1 to 3 GHz. It can be seen that the measured input reflection coefficient is less than −8 dB, which is 3 dB lower than the simulated one. The measured small-signal gain is better than 19.8 dB, which is 2 dB lower than the simulated one. The discrepancy between the measured and simulated S-parameters is mainly caused by the SMA connector at the input and output of the measurement fixture and the inaccuracy of the GaN active device model.

### 4.2. Large-Signal Measurement

**Figure 12:**
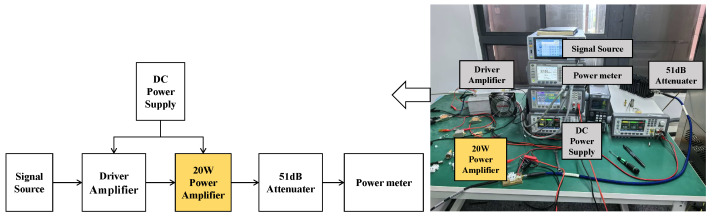
Large-signal parameter measurement setup.

Figure 12 is the picture of the large-signal parameter measurement setup. Vector Signal Generator SMCV100B from Rohde & Schwarz (Munich, Germany) is applied to generate the RF signal. A Driver amplifier is applied to provide enough input power for DUT. Power Meter N1912A from Keysight (Santa Rosa, CA, USA) is applied to measure the accurate output power of DUT. The DC current is measured by the DC power supply. A continuous wave (CW) is implemented to test the performance of the proposed PA. The input power range is from 14 dBm to 28 dBm. The operation frequency is 2.45 GHz.

**Figure 13:**
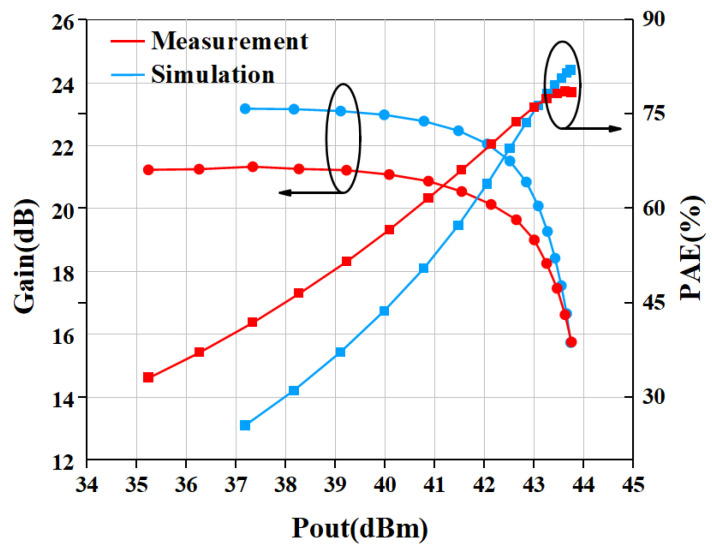
Simulated and measured large-signal performance with harmonic tuning circuit.

**Figure 14:**
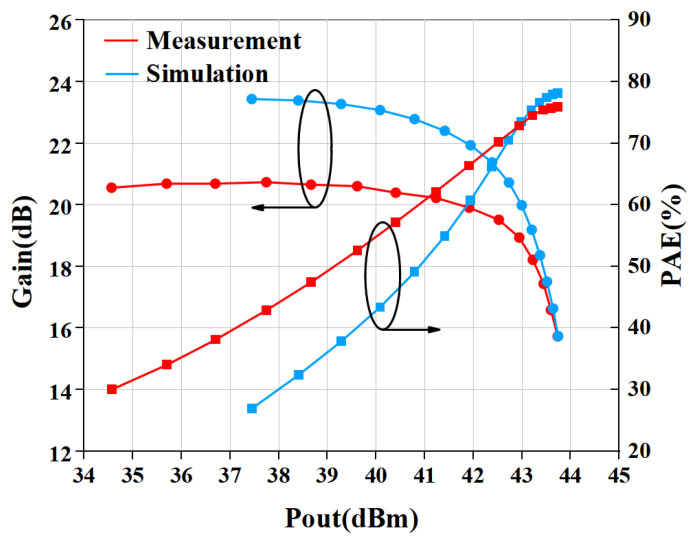
Simulated and measured large-signal performance without harmonic tuning circuit.

**Figure 15:**
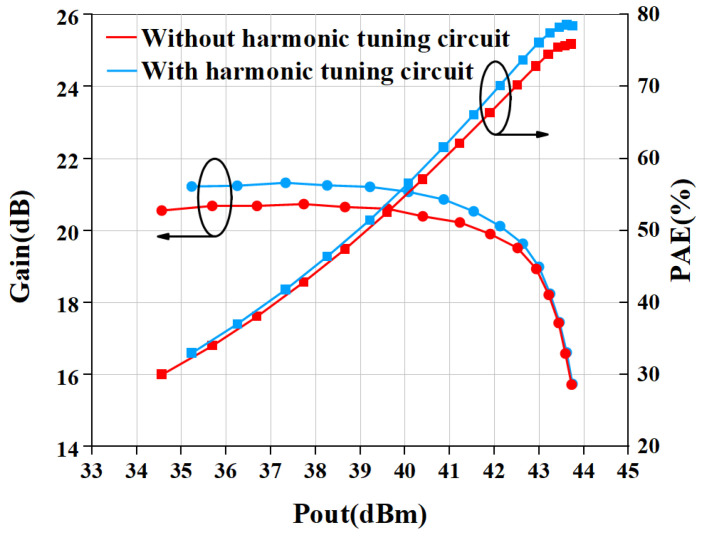
Measured large-signal performance with and without harmonic tuning circuit.

**Figure 16:**
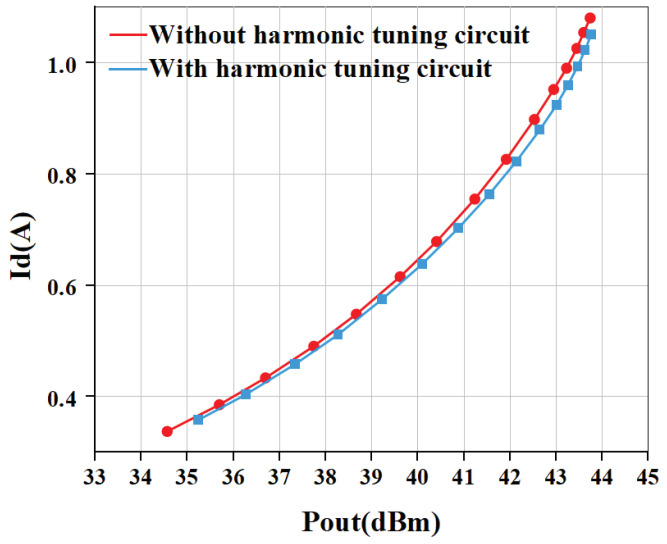
Comparison of measured dynamic drain current in two cases.

Figure 13 gives the comparison result of measured and simulated data of the proposed PA. The gain and PAE curves are shown in Figure 13. The saturated power of the internally-matched PA is 43.75 dBm (23.71 W), while the PAE is 78.5% when input with 28 dBm power. The gain is 21.2 dB in the linear range and 15.75 dB at saturation power. The $P_{1dB}$ is 42.13 dBm. In addition, we also measured the output power and PAE of the power amplifier without an input harmonic tuning circuit, as shown in Figure 14. The measurement of PA without a harmonic tuning circuit is done after removing the bonding wire of the input harmonic tuning circuit. When the input power is 28 dBm, the output power of the power amplifier is 43.73 dBm (23.6 W) while the PAE is 76.0%. This indicates that the PA is still in a high-efficiency mode, which means the output harmonic load is much more important than the input harmonic load. In our design, the output matching has already satisfied the output harmonic load condition so that the PA can perform well without input harmonic tuning. It can be seen from Figure 15 that the PAE of the harmonic tuning circuit is 2.5% higher than that of the non-harmonic tuning circuit under the same output power, which is mainly due to the reduction in the dynamic drain current as shown in Figure 16. In addition, it can be seen that there is a certain gap between the measured result and the simulated result. From Figure 13 and Figure 14, the simulation results with harmonic tuning have a larger gap with measured results than the simulation without harmonic tuning. And, all the measured results show lower output power when the input power is low. This indicates that the harmonic load of the GaN model is not correct in the low-power region. It can be noticed that the simulated PAE is lower than the measured results; the inaccuracies in the Pdc representation within the GaN model contribute to these issues. Additionally, discrepancies between the modeling test environment and the application test environment lead to variations in simulated Pdc. This has a significant influence on PAE and gain simulation in low-power regions.

(5) $$Q_{di}=P_{out}\left(W\right)\cdot \mathrm{PAE}/\mathrm{Dimension}\left({\mathrm{mm}}^{2}\right)$$

Table 1 displays the comparison of the latest advancements in correlation design performance. We define $Q_{di}$ which is shown in (5) to compare the performance of each power amplifier. It can be seen from Table 1 that most of the externally-matched PA has good PAE performance at 2.45 GHz. However, the circuit size of externally matched PAs is much larger than this work. Although MMIC PAs have a smaller circuit size, our work performs with better power and PAE. Our work can achieve 23-watt output power with a circuit size of 13.4 × 13.5 mm^2^, which makes it suitable for wireless power transfer systems.

## 5. Conclusions

In order to meet the high-power, high-efficiency, and miniaturization requirements of a microwave wireless power transfer system, a high-power and high-efficiency internally matched power amplifier based on an off-chip LC tuning circuit at 2.45 GHz is designed in this paper. The second and third harmonic matching impedance can be reached without an extra harmonic circuit, which simplifies the output matching design and reduces the circuit size. Further more, the input harmonic tuning circuit has the advantages of a moderate circuit area, excellent harmonic control ability, and low loss. Based on the proposed method, an internally-matched power amplifier at 2.45 GHz is fabricated by using a 0.25 μm GaN HEMT. The results show that output power is 43.75 dBm, large-signal gain is over 15.75 dBm, and PAE is 78.5% at 2.45 GHz. The proposed PA is implemented with a size of 13.4 × 13.5 mm^2^. The compact size, high efficiency, and high power can allow the PA in this work to potentially be usefed in wireless power transfer systems.

## Figures and Tables

**Table 1:** Comparison of state-of-the-art PA under CW operation.

Reference	Freq (GHz)	Pout (W)	Gain (dB)	PAE (%)	Type	$Q_{\mathrm{di}}$	Size (mm^2^)
2018 [14]	2.45	450	13	67	Internally-matched	NA	NA
2020 [24]	2.4	33	13.6	78.8	Externally-matched	1.27	31×66
2013 [25]	3.1	10	15	82	Externally-matched	0.46	40×45
2009 [26]	3.5	11	12	78	Externally-matched	0.1	80×110
2021 [27]	2	7	10	74	Externally-matched	0.17	51×59
2023 [28]	4.25	11.8	11.5	55.3	Externally-matched	0.14	62×77
2022 [10]	2.21	11.5	15.6	82.6	Externally-matched	0.51	29×64
2022 [29]	1.97	10.4	10.8	79.3	Externally-matched	0.69	26×46
2024 [30]	2.6–3.6	12.0	8.5	50.8	MMIC	45.8	3.5×3.8
2021 [31]	2.6–3.8	5.25	10.2	55	MMIC	29.6	6.5×1.5
This work	2.45	23.7	15.75	78.5	Internally-matched	10.28	13.4×13.5

## Data Availability

The data presented in this study are available in this article.
